# Material Culture: Still ‘Terra Incognita’ for Psychology Today?

**DOI:** 10.5964/ejop.v11i2.995

**Published:** 2015-05-29

**Authors:** Christiane Moro

**Affiliations:** a Institute of Psychology, University of Lausanne, Lausanne, Switzerland

Hamlet: Do you see nothing there?

Gertrude (The Queen): Nothing at all, yet all that is I see.

HAMLET, Act III, Scene IV

Although over the past decades culture can be said to have re-entered psychology, one can only be puzzled by the fact that material culture did not receive the same attention and has been neglected by both ‘mainstream’ and cultural-historical traditions. To counter-balance this strange absence, the assumption I start from in this editorial is that material culture and material objects are at the heart of human developmental processes, conditioning our everyday relationship to the world, and influencing how we live, act, think and develop as human beings from an early age. This view bridges the gap between what is traditionally identified as, on the one hand, symbolic and, on the other, material culture. In psychology, on-going scientific work is being undertaken to unpack the role of material culture for the developing subject and the way in which it elicits and transforms his/her psychological processes in a dialectical movement of appropriation and transformation of culture through innovation. This emerging field extends the psychological to the mundane world, which becomes an essential player in the area of psychological development.

Considering the fact that our daily lives are characterised by innumerable encounters with material objects that obviously, in a way or another, organise our relationship to the world, the very first question is why psychology has been so silent concerning this crucial issue and why it has taken so long to inscribe material culture into its scientific agenda. Even in the emerging stream of research in psychological development where materiality is now being considered a crucial actor, a second very important question arises: how can we progress in the problematisation of material culture and its objects for a better understanding of the evolution and transformation of psychological processes?

Why such a lack of interest for material culture in psychology? In ‘mainstream’ psychology and in the field of developmental psychology, *the reality* (also called *the real*) refers to an *objective world* that the subject *thinks* about. Inaugurated by Piaget’s (1952, 1954) seminal research, this conceptualisation of the object refers to it as ‘what is placed in front of’, ‘which exists independently from the mind’ (from the Latin *objectum*). It conceives objects in terms of their physical properties, as a result of an attribution by the solitary subject. This view is grounded in the classical subjectivity orientation within philosophy, inspired by Descartes and Kant, in which the world is theoretically defined as an objective entity. From that time onwards, the object has been limited to the rational and consequently, its historical, cultural and semiotic features have been overlooked or naturalised. To come back to the example of early development, besides Piaget, this is also the case for subsequent inneist, computationalist and social cognition perspectives (e.g., Baillargeon, Spelke, & Wasserman, 1985; Leslie, 1987, inspired by Fodor; and Tomasello, 1999, inspired by Gibson).

The same surprising absence applies, but for different reasons, to the cultural-historical developmental tradition which has some difficulties in placing the issue of material culture on its agenda. Why such a lack of consideration of the cultural status of the object in the cultural-historical framework? Representing this tradition, Vygotsky (1962; Vygotsky & Luria, 1994) attributes a considerable role to language, considered as the semiotic system *par excellence* in human development. We assume that this pre-eminence of language is the consequence of a focus on social relations grounded in the legacy of Marx’s anthropology (cf. *The 6th Thesis on Feuerbach*) where it is asserted that the *humanitas de l’homo*, i.e., “[…] the human essence is no abstraction inherent in each single individual. In its reality it is the ensemble of *social relations*” (Marx, 1845 [my emphasis]). If we do agree with this conception of external human essence as expressed by Marx, we consider that the identification of the culture to the social in Vygotsky's framework leads to neglecting the intrinsic meaning of the object as related to its conventional use in favour of an extrinsic meaning, over-determined by the linguistic device.

As a consequence, in the above positions, the object is invisible and the role of material culture for human development is under-theorised. The aim of making visible material culture in psychology brings us close to the field of *Material Culture Studies* (e.g. Hicks & Beaudry, 2010; Tilley, Keane, Küchler, Rowlands, & Spyer, 2006) in which materiality is a growing research topic emerging at the frontier between archaeology and anthropology. This new, interdisciplinary field, unbounded and unconstrained, reconsiders material culture as “an integral dimension of culture, and that there are dimensions for social existence that cannot be fully understood without it” (Tilley et al., 2006, p. 1). The domain of things or objects is the principal concern but, alternatively, material culture studies can also take the human subject or the social as their starting point (Tilley et al., 2006). This field of research is animated by substantive debates and, amongst them, the utility of creating a separate category of the ‘material’ that is not materially enacted (Hicks, 2010, referring to Ingold, 2007). This question is intrinsically linked to the definition of material culture and material objects, even if some scholars refuse to consider it (see Miller, 2010).

In psychology, an increasing stream of interdisciplinary work dealing with objects, things, artefacts, etc., reflecting various orientations of research and using different conceptual and theoretical frameworks one can identify. These works broadly originate in cultural-historical perspectives without excluding the interconnection with other psychological traditions such as (without being exhaustive) cognitive perspectives, biological theory, neurosciences, etc., and making use of semiotic, phenomenological, ecological or anthropological approaches. These studies are trying to reflect on what was a taken-for-granted issue, insisting on the significance and importance of investigating material domains to understand human development (e.g., Andrén, 2010; de La Ville & Tartas, 2010; Glăveanu, 2014; Moro, 2011; Moro & Muller Mirza, 2014; Moro & Rodríguez, 2005; Muller Mirza & Perret-Clermont, 2008; Rickenmann, 2014; Sinha, 2005) with topics concerning, for example, early development, creativity, the work of art, consumption, education, professional practices, gestures, among others.

In this strive to reintroduce materiality in psychology and to explore its cultural, historical and semiotic status in human development, it is interesting to turn, once again, to philosophy. In particular towards phenomenology and to Heidegger who challenged the Kantian *doxa* by reintroducing a reflection on things and objects in the ordinary world. His perspective is filling, in a certain way, the gap left opened in cultural-historical theory (cf. Vygotsky) concerning the material world. We choose to address this approach, in the end, as it is an illustration of the perspective of ordinariness also evoked by Schütz and Luckmann (1973) and Searle (1995) and because that it might constitute a new and fruitful perspective for psychology (cf. also Wittgenstein, 1961 and Cavell, 1986).

Challenging the Kantian *doxa* by rethinking the world and things in their ordinariness – within which, since the beginning, human relationships are embedded – the conception of phenomenology sheds new light on the issue of ‘Wordliness’ of the world through, inter alia, a reflection on the ‘Thing’. In *Being and Time*, Heidegger (1996) goes beyond Kant’s classical subjectivity by exploring the Wordliness of the world in order to understand what it means to be a world for the *Dasein*. Etymologically, *Dasein* (German neologism) means ‘Being-there’ and is usually translated by ‘Being-in-the-world’. What is interesting for the question of materiality is that the world is reintroduced in its ordinariness and is considered as a significant whole in relation to the Dasein. The world in which one dwells may be accessed through activities, by how one engages with things in the world in a pragmatic mode. This contrasts with Kant’s *doxa*, where the world is only accessible in the mode of knowing. As an example, in *The Thing*, Heidegger (1971) is interested in that sort of things of the world that can be used such as a jug. He takes the example of a handmade ceramic jug and asks ‘what is a jug?’ in its fundamental Thingness. The jug then can be defined through the void inside it whose basic function is ‘to hold’, which is the *way of being* of the jug for the Dasein. The advantage of engaging with Heidegger’s views is that we are immersed into the world of things; in it, we live, encounter and experience things through how we perceive and use them ordinarily.

The reflexion on the Worldliness of the world provides new insight on the issue of materiality by making us aware of the conventional use of things. Pursuing cultural-historical perspectives which focus on explaining the practical and historical relationship of the developing subject to the world, one of the questions raised is how this world and its realities are appropriated by the subjects and how psychological processes are elaborated thanks to this appropriation, considering that these relationship are constantly re-qualified by the developing subject in the course of his/her interactions with the world and other people. Here the question of meaning is crucial to consider. Material culture has a double nature. It is material but it is also a site of public meanings through the conventional uses of objects. How people access these public meanings and how do they transform them? How to understand then the question of affordances? As a starting point or as the point of arrival for the reconstruction of the intrinsic meaning of the object? And how to redefine the question of intersubjectivity? How to speak of meaning-making processes in relation to the activity of the subject concerning the object? And how to link these meanings to other forms of meanings, including those concerning corporeity and language, in a multimodal perspective of meaning-making processes?

The reintroduction of the material world of culture (and conventions) at the very core of the object (or the thing), by contrasting classical subjectivity, where objects are reduced to the subjectivity and representation, increases the complexity of our relationship with the world and with other people, making obvious the new challenges introduced in the study of human development thanks to materiality.
